# Clinical analysis of decision implementation by a multidisciplinary team in cervical cancer cases in Ganzhou, China

**DOI:** 10.3389/fonc.2023.1160626

**Published:** 2023-08-17

**Authors:** Jing Huang, Xueqin Zeng, Hailong Chen, Deping Luo, Rong Li, Xiuhong Wu, Ying Yu, Ailin Chen, Chan Li, Yiyun Pan

**Affiliations:** ^1^ Department of Gynecology and Oncology, Ganzhou Cancer Hospital, Jiangxi, China; ^2^ Department of Institute of Cancer Research, Ganzhou Cancer Hospital, Jiangxi, China; ^3^ Department of Chemotherapy Center, Ganzhou Cancer Hospital, Jiangxi, China; ^4^ Department of Pathology, Ganzhou Cancer Hospital, Jiangxi, China; ^5^ Department of Radiotherapy Center, Ganzhou Cancer Hospital, Jiangxi, China; ^6^ Department of Image Center, Ganzhou Cancer Hospital, Jiangxi, China

**Keywords:** cervical cancer, multidisciplinary team, precise treatment, clinical practice, multidisciplinary diagnosis and treatment of cancer

## Abstract

**Objective:**

In this study, we evaluated the role of a multidisciplinary team (MDT) in clinical practice for cervical cancer by analyzing the development of a single-case multidisciplinary consultation for cervical cancer.

**Methods:**

Patients in MDT consultations for cervical cancer were retrospectively analyzed for clinical information, decision content of MDT discussion, implementation, and follow-up results.

**Results:**

Of the 392 patients who met the inclusion criteria, 359 had a first episode, of which 284 were stage IA-IIA2 (79.11%) and 75 were stage IIB-IVB (20.89%). Of these 392, 33 had a recurrence (8.42%). A total of 416 cases were analyzed, and neoadjuvant chemotherapy with surgery was recommended in 43 cases, of which 40 cases were implemented, and 36 of the 40 achieved the expected outcome. Surgical treatment was recommended in 241 cases, of which 226 underwent surgery, and 215 of them achieved the expected outcome. Radiotherapy was recommended in 31 cases, of which 26 cases underwent it, and 22 of them achieved the expected efficacy. Concurrent chemoradiotherapy was recommended in 57 cases, of which 49 underwent it, and 39 of them achieved the expected efficacy. Other treatments were recommended in 44 cases, of which 23 cases were implemented, and 10 of them achieved the expected efficacy, with statistically significant differences compared with cases without implementation (*P <*0.05). MDT decisions were correlated with age; the younger the patients, the higher the implementation efficiency (*P <*0.05). The difference between MDT expectation in all implementation and partial implementation and age was statistically significant (*P <*0.05). No significant difference was found between age and MDT expectation in all not fully implemented decisions (*P >*0.05). Some decisions were not fully implemented due to economic status and fear of certain treatments of the patient.

**Conclusion:**

The MDT plays an important role in clinical practice such as clinical staging, treatment plan, and the complete treatment management of patients with cervical cancer, which can significantly improve the near-term treatment effect, whereas its effect on a long-term prognosis needs further clinical observation and active exploration.

## Introduction

Cervical cancer is the most common malignant tumor in gynecology. Each year, more than half a million women are diagnosed with cervical cancer, and the disease results in over 3,00,000 deaths worldwide. More than 85% of new cases and approximately 90% of deaths occur in developing countries (1, 2). Whether treatment should be surgery or radiotherapy is closely related to cervical cancer staging, which is discussed and decided by gynecologists, radiologists, imaging physicians, and pathologists (3, 4). Extensive hysterectomy combined with complete pelvic lymph node dissection for cervical cancer requires the assistance of anesthesiology, colorectal, vascular, urological, reproductive, pathology, nutrition, and intensive care units as well as nursing teams (5–7). Radiotherapy for cervical cancer involves oncologic radiotherapy, oncologic chemotherapy, gynecologic oncology, pathology, nutrition, and nursing teams (8, 9). Cervical cancer treatment requires multidisciplinary communication and collaboration to develop individualized and high-quality precise treatment plans to improve the diagnosis and treatment of cervical cancer and improve the survival and quality of life of these patients (10, 11).

Multidisciplinary collaboration for cervical cancer treatment needs to integrate the clinical knowledge, skills, and experience of all relevant departments in a hospital or relevant multidisciplinary team (MDT) models in higher-level hospitals according to a patient’s physical and psychological conditions, pathological type, clinical stage, and the developmental tendency for high-quality diagnosis and a precise individualized treatment plan based on evidence-based medicine to achieve the best treatment outcome that is economical and can maximize the survival rate and quality of life (12, 13). For complex and difficult cases, the MDT approach should be used to recommend individualized and precise treatment plans on the basis of a patient’s wishes and affordability, which are guided by the latest research and guidelines, and one should master the appropriate timing of treatment and prevention of complications (14, 15). A few studies on MDTs for cervical cancer are available. Thus, in this study, we evaluated the role of an MDT for cervical cancer in clinical practice by analyzing the clinical data of MDT consultation, decision content of MDT discussion, implementation, and follow-up results of patients with cervical cancer.

## Materials and methods

### Ethics statement

The present study was approved by the Ethical Committee of Ganzhou Cancer Hospital (2023002), and written informed consent was obtained from participants. The study was performed in accordance with the principles of the Declaration of Helsinki regarding research involving human subjects. Each patient provided written informed consent to participate after the nature of the study was explained to them.

### Study design

A retrospective analysis was performed on the development of multidisciplinary consultation for patients with cervical cancer who fulfilled the inclusion criteria. The role of MDT in the clinical practice of cervical cancer was evaluated.

Patient inclusion criteria, patients with (i) pathologically confirmed cervical cancer; (ii) first diagnosis or previous treatment; (iii) at least one MDT discussion; and (iv) complete clinical data were included in the study. Patient exclusion criteria, (i) patients with cervical cancer who did not participate in MDT discussions; (ii) patients with incomplete primary study information; and (iii) patients who were lost to follow-ups were excluded from the study.

### Participants and setting

Ganzhou Cancer Center/Ganzhou Cancer Hospital in China is a large-scale, tertiary-level, A-class specialized oncology hospital. In total, 392 patients with cervical cancer from the Department of Gynecology and Oncology of Ganzhou Cancer Center/Ganzhou Cancer Hospital from January 2017 to December 2020 who met the inclusion criteria were selected. We included 83 cases (21.17%) from 2017, 95 cases (24.24%) from 2018, 104 cases (26.53%) from 2019, and 110 cases (28.06%) from 2020. with 359 cases of first presentation, including 284 cases of stage IA-IIA2, 75 cases of stage IIB-IVB, and 33 cases of recurrence. The age ranged from 23 to 70 years, with a median age of 52.46 years. A total of 416 MDT discussions were conducted in all patients (**Table 1**).

**Table 1:** A retrospective cohort of 392 clinical cases.

Clinical Information	Composition ratio, n (%)
Age (years)	
20–40	47 (11.99)
41–50	132 (33.67)
50–60	115 (29.34)
61–70	98 (25.00)
KPS	
≥80	258 (65.82)
<80	134 (34.18)
Other concomitant diseases	
Yes	99 (25.26)
No	293 (74.74)
Pathological type	
Squamous cell carcinoma	362 (92.35)
Adenocarcinoma	21 (5.36)
Adenosquamous carcinoma	9 (2.29)
Pathological grade	
G1	24 (6.12)
G2	242 (61.73)
G3	117 (29.85)
Gx	9 (2.30)
FIGO stage	
IA-IIA2	284 (72.45)
IIB~IVB	75 (19.13)
No stage of recurrence	33 (8.42)
Initial treatment	
Yes	359 (91.58)
No	33 (8.42)

### Characteristics of the MDT

**Figure 1:**
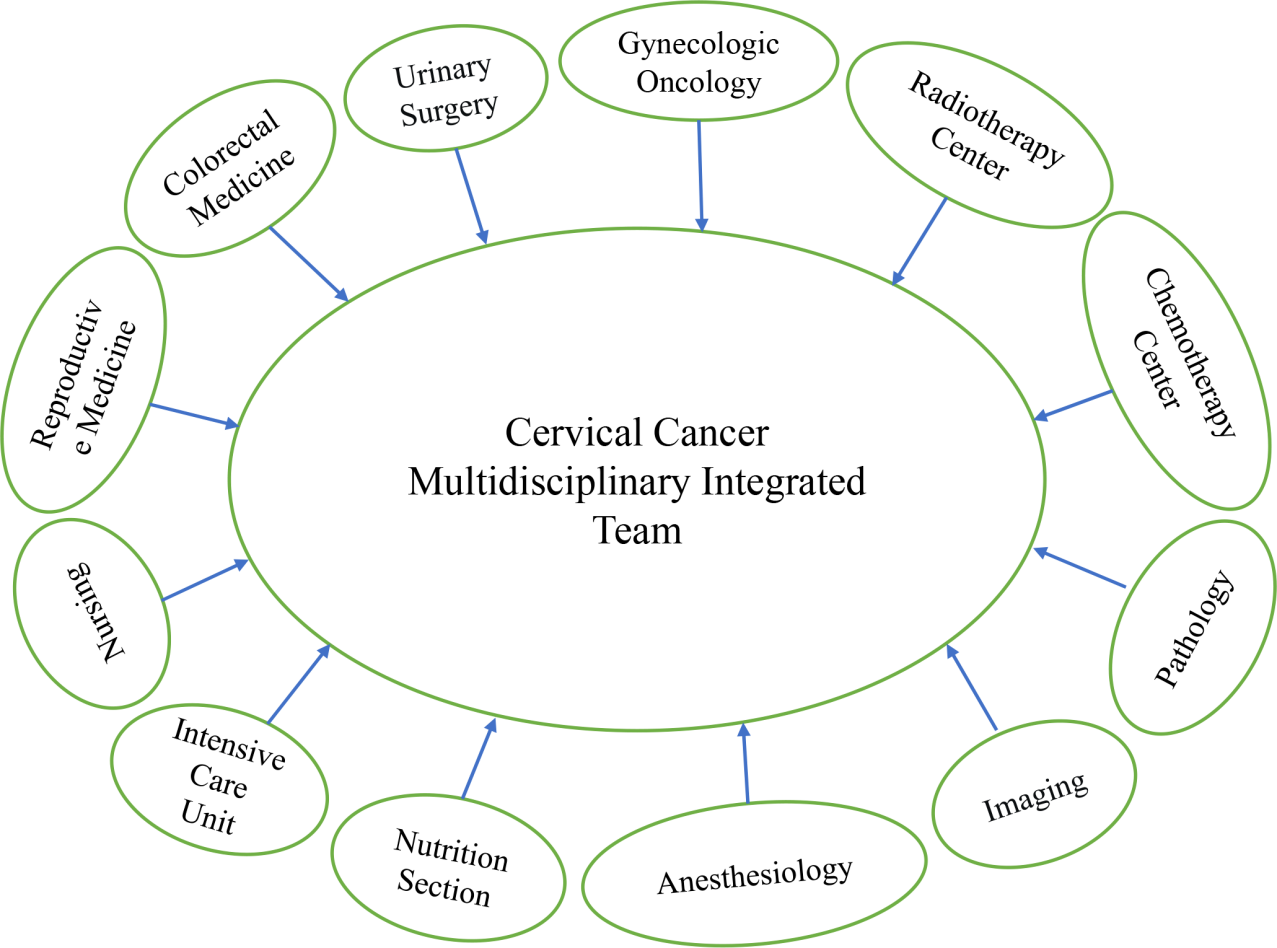
Cervical cancer MDT consultation team model.

Since its establishment in 2012, the cervical cancer MDT treatment at the Ganzhou Cancer Center has gradually improved and developed to maturity by 2016. The MDT consisted of the Department of Gynecologic Oncology as the core, the Radiotherapy Center and Chemotherapy Center as the backbone, along with the Departments of Urology, Colorectal Medicine, Critical Care Medicine, Anesthesiology, Imaging, Pathology, and Nutrition. **Figure 1** shows the comprehensive treatment team formed for patients with cervical cancer, especially those with complicated cases of cervical cancer. The chief expert is the head of the Department of Gynecologic Oncology, a chief physician or professor, and the other team members were the head of the department, associate chief physicians, or associate professors or above.

**Figure 2:**
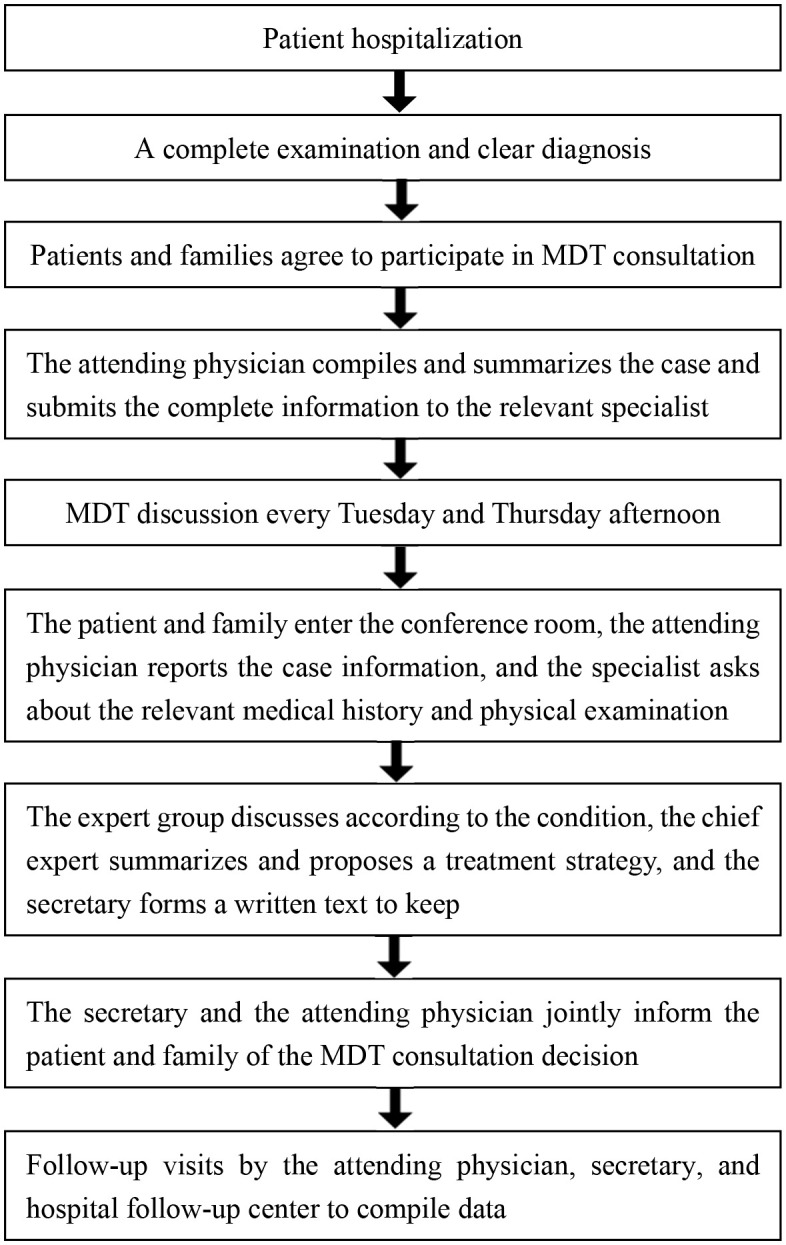
Cervical cancer MDT consultation process.

Patients are admitted to the hospital after the suspicion or diagnosis of cervical cancer. All tests are performed to clarify the diagnosis; patients’ conditions, the MDT treatment concept, and its advantages are explained clearly to these patients and families, and an attending physician submits an MDT discussion after consent. An expert group further discusses these conditions, a chief specialist summarizes and proposes a treatment strategy, a secretary maintains a written record, and the secretary and attending physician jointly inform the patients and families of the MDT consultation decision (**Figure 2**). When the experts in the group have a disagreement, the chief expert will create individualized and precise treatment plans based on the clinical skill and experience of the team, evidence-based medicine, and the wishes and financial status of the patient. The MDT for cervical cancer from a higher hospital can be contacted to conduct a discussion together with our MDT via a video call to create the most accurate individualized treatment plan. For difficult cases, patients can contact the cervical cancer MDT of a higher hospital, submit the complete information to MDT experts of the higher hospital, and conduct an MDT discussion with the cervical cancer MDT of the hospital via video conference.

The MDT meeting is usually held in the conference room of the Department of Gynecologic Oncology or the hospital teleconsultation center and is attended by members of the MDT specialists, new doctors, and students from the gynecologic oncology department, chemotherapy center, and radiotherapy center. They discuss two to three patients at a time during 1–1.5 hours. After creating the treatment plan, the Department of Gynecologic Oncology, the Radiotherapy Center, and the Chemotherapy Center are responsible for its implementation.

### Assessment of decision implementation

The 392 patients were followed up until June 2022; the follow-up period was of 7–65 months, and the mean follow-up period was of 25.6 months. The mean follow-up time was 29.6 months for the 20–40 age group, 28.4 months for the 41–50 age group, 27.1 months for the 51–60 age group, and 18.2 months for the 61–70 age group. The patients were followed up by the attending physician, MDT secretary, and hospital follow-up center according to the principles of oncology follow-up. The outpatient follow-up was performed by the attending physician, whereas the telephone follow-up was performed by the MDT secretary and hospital follow-up center. All MDT consultation decisions completed are “fully implemented”, those partially completed are “partially implemented”, and those not implemented are “not implemented”. The implementation status of the MDT consultation decision for each patient was analyzed, and the implementation effect was recorded.

Implementation outcomes were classified as “meeting expectations” and “not meeting expectations”. Expectations were met when the diagnosis was confirmed by MDT discussion, radical resection was achieved by surgery, radical treatment was achieved by radiotherapy, and symptom improvement or disease control was achieved by palliative care. The “not meeting expectations” outcomes included no confirmed diagnosis after MDT discussion, tumor residual after surgery or radiotherapy, and failure to improve symptoms or prolong survival after palliative care.

### Statistical analysis

Statistical analysis was performed using IBM SPSS Statistics for Windows, version 21.0. Data are expressed as percentages. A hypothesis testing model was constructed for classified data. The χ^2^ test was performed to compare statistical data between the groups, and P < 0.05 was considered statistically significant.

## Results

### MDT consultation discussion

The MDT consultation was discussed in 416 cases for 392 patients, of which 16 cases attended the consultation twice and four cases attended thrice, and repeat MDT consultations accounted for 5.1%. All the 416 MDT consultations involved diagnosis and treatment, and patients attended MDT consultations to know accurate cancer staging and treatment strategies. Among them, neoadjuvant chemotherapy with surgery was recommended in 43 cases (10.34%), surgery was recommended in 241 cases (57.93%), radiotherapy was recommended in 31 cases (7.45%), simultaneous radiotherapy and chemotherapy were recommended in 57 cases (13.70%), and other treatments were recommended in 44 cases (10.58%). Chemotherapy was recommended in 15 cases (3.61%), chemotherapy with targeted therapy was recommended in 13 cases (3.13%), chemotherapy with immunotherapy was recommended in 11 cases (2.64%), and targeted therapy with immunotherapy was recommended in five cases (1.20%) for other treatments (**Tables 2**, **3**). Postoperative patients were treated by gynecologic oncologists, pathologists, and radiologists who discussed the postoperative treatment plan and recommended observation, radiotherapy, or concurrent radiotherapy. Moreover, all patients underwent psychotherapy, including supportive de-escalation, emotional rationalization, group therapy and relaxation therapy, and individualized nutritional support treatment.

**Table 2:** Multidisciplinary team decision-related content (n = 416).

Decision content	Composition ratio, n (%)
Neoadjuvant chemotherapy + surgery	43 (10.34)
Surgery	241 (57.93)
Radiotherapy	31 (7.45)
Concurrent chemoradiotherapy	57 (13.70)
Other treatments	
Chemotherapy	15 (3.61)
Chemotherapy + targeted	13 (3.13)
Chemotherapy + immunization	11 (2.64)
Targeted + immunization	5 (1.20)

**Table 3:** Multidisciplinary team decision-making and age-related content (n = 416).

Age (years)	Decision content n (%)				
	Neoadjuvant chemotherapy + surgery	Surgery	Radiotherapy	Concurrent chemoradiotherapy	Other treatments
20–40	5 (10.64)	35 (74.47)	3 (6.38)	4 (8.51)	0 (0.00)
41–50	13 (9.35)	93 (66.91)	11 (7.91)	17 (12.23)	5 (3.60)
50–60	15 (12.10)	70 (56.45)	8 (6.45)	20 (16.13)	11 (8.87)
61–70	10 (9.43)	43 (40.57)	9 (8.49)	16 (15.09)	28 (26.42)

### MDT decision implementation

Of the 416 MDT decisions, 283 (68.03%) were “fully implemented”, 81 (19.47%) were “partially implemented”, and 52 (12.50%) were “not fully implemented” (**Table 4**). Some decisions were not fully implemented due to the economic status and fear of certain treatments of the patients (**Table 5**). MDT decisions correlated with age; the younger the patients, the higher implementation efficiency (χ^2^ = 11.2286, P = 0.0106) (**Table 6**). In 16.59% (40/241) of cases, the MDT decision was a joint operation between two specialties, all of which sought intraoperative assistance. Eight cases of injury to the internal iliac vein were repaired by vascular surgery, and four cases of injury to the bowel were repaired by colorectal surgery. Six cases of intraoperative bladder injury, 10 cases of ureteral injury, and 28 cases of ureteral stent placement. Urology (7.14%) was the specialty that cooperated most with cervical cancer surgery (**Table 7**).

**Table 4:** Multidisciplinary team decision implementation (n = 416).

Parameter	Composition ratio, n (%)
All execution	283 (68.03)
Partial execution	81 (19.47)
All not executed	52 (12.50)

**Table 5:** Reasons for Partial execution and all not executed n (%).

Reasons	Composition ratio, n (%)
Partial execution	
Economic reason	35 (43.21)
Fear of a certain treatment	42 (51.85)
Other reasons	4 (4.94)
All not executed	
Economic reason	35 (67.30)
Fear of a certain treatment	15 (28.85)
Other reasons	2 (3.85)

**Table 6:** Multidisciplinary team decision-making execution and age-related content, n (%) (n = 416).

Age (years)	Execution	Not executed	χ^2^	*p*
20–40	46 (97.87)	1 (2.13)	11.2286	0.0106
41–50	126 (90.65)	13 (9.35)		
50–60	107 (86.29)	17 (13.71)		
61–70	85 (80.19)	21 (19.81)		

**Table 7:** Multidisciplinary team decision combined with surgery (n = 392).

Combined surgery	Composition ratio, n (%)
Vascular surgery	8 (2.04)
Colorectal medicine	4 (1.02)
Urologic surgery	28 (7.14)

### Efficacy assessment of MDT decisions

The efficacy of decisions made by MDT was evaluated in 416 cases. In the 364 cases (87.50%) of full and partial implementation, 325 (89.29%) cases met MDT expectations. The expected efficiency of full implementation was 93.99% (266/283), and the expected efficiency of partial implementation was 72.84% (59/81), with a statistically significant difference (χ^2^ = 29.4569, P = 0.0000) (**Table 8**). The difference between MDT expectation in all implementation and partial implementation and age was statistically significant (χ^2^ = 10.4283, P = 0.0153) (**Table 9**). Full non-implementation was achieved in 52 cases (12.50%) and expected efficacy was achieved in 20 cases (38.46%). No significant difference was found between age and MDT expectation in all not fully implemented decisions (χ^2^ = 11.1744, P = 0.7592) (**Table 10**).

**Table 8:** Efficacy assessment for MDT decision-making, n (%) (n = 416).

MDT decision-making	Meet expectations	not meet expectations	χ^2^	*p*
Neoadjuvant chemotherapy + surgery			7.2901	0.0069
Execution	36 (90.00)	4 (10.00)		
Not executed	1 (33.33)	2 (66.67)		
Surgery			18.3132	0.0000
Execution	215 (95.13)	11 (4.87)		
Not executed	10 (66.67)	5 (33.33)		
Radiotherapy			8.8495	0.0029
Execution	22 (84.62)	4 (15.38)		
Not executed	1 (20.00)	4 (80.00)		
Concurrent chemoradiotherapy			6.1735	0.0130
Execution	39 (79.59)	10 (20.41)		
Not executed	3 (37.50)	5 (62.50)		
Other treatments			4.7489	0.0203
Execution	13 (56.52)	10 (43.48)		
Not executed	5 (23.81)	16 (76.19)		

Other treatments include chemotherapy, chemotherapy + targeted therapy, chemotherapy + immunotherapy, and targeted + immunotherapy.

**Table 9:** Efficacy assessment and age of full and partial implementation MDT decisions, n (%) (n = 364).

Age (years)	Meet expectations	Not meet expectations	χ^2^	*p*
20–40	43 (93.48)	3 (6.52)	10.4283	0.0153
41–50	119 (94.44)	7 (5.56)		
50–60	94 (87.85)	13 (12.15)		
61–70	69 (81.18)	16 (18.82)		

**Table 10:** Efficacy assessment and age of full non-implementation MDT decisions, n (%) (n = 52).

Age (years)	Meet expectations	Not meet expectations	χ^2^	*p*
20–40	0 (0.00)	1 (100.00)	1.1744	0.7592
41–50	4 (30.77)	9 (69.23)		
50–60	7 (41.18)	10 (58.82)		
61–70	9 (42.86)	12 (57.14)		

## Discussion

Patients undergoing monotherapy require multiple referrals or consultations, which can be time-consuming and result in poor adherence to treatment. A treatment model via an MDT integrates the advantages of various related disciplines to develop an individualized, high-quality, and precise treatment plan, which plays an important role in improving the survival rate, quality of life, and prognosis of patients (10–15). In this study, we evaluated the role of an MDT in clinical practice for cervical cancer by analyzing the clinical data of MDT consultation, decision content of MDT discussion, implementation, and follow-up results of patients with cervical cancer.

All 392 patients were unvaccinated. Among them, 65.82% had a KPS score of ≥ 80 at admission, 359 had a first episode, including 284 in stages IA–IIA2, 75 in stages IIB–IVB, and 33 had a relapse, with a minimum of 2 days and a maximum of 2.5 months from diagnosis to treatment. We analyzed the clinical data of 416 MDT cases of cervical cancer and compared the outcomes of 364 cases with implemented decisions and the 52 cases without implemented decisions. We found that patients with implemented decisions were significantly better than those without implemented decisions in terms of diagnosis time, surgery efficiency, standardized radiotherapy, standardized chemotherapy, and other treatments. Moreover, the expected efficiency of full implementation was higher than that of partial implementation (93.99% vs. 72.84%), with a statistically significant difference (*P* < 0.05). Of note, 16.59% of patients with internal iliac vein injury, bowel injury, bladder injury, ureteral injury, and intraoperative placement of ureteral stents were joint surgeries between two specialties with adequate preoperative evaluation. Urology was the specialty with the highest cooperation with cervical cancer surgery. The decisions of all 416 cases involved diagnosis plus treatment, and treatment decisions included neoadjuvant chemotherapy plus surgery, surgery, radiotherapy, concurrent chemoradiotherapy, and other treatments. Other treatments included chemotherapy, chemotherapy plus targeted therapy, chemotherapy plus immunotherapy, and targeted plus immunotherapy. All postoperative outcomes of surgical patients were discussed by gynecologic oncologists, pathologists, and radiotherapists on the basis of the postoperative treatment plan, and observation, radiotherapy, or concurrent radiotherapy was recommended. Furthermore, symptomatic treatments, such as nutrition and psychological support, in MDT consultation can alleviate the symptoms of the patients, reduce their discomfort, and improve their quality of life.

Maturation and close collaboration of MDT for cervical cancer requires a long-term process (16). Decisions made by the MDT may not be accurate, and even after maturation, the team members can change, which can result in poor collaboration. Moreover, the MDT of one center may not be able to make an individualized treatment plan in the best interest of the patient during difficult cases because of its platform and learning. Therefore, they may often require the assistance of the MDT for cervical cancer in a higher, larger medical center to discuss the condition of the patient via a video conference by two different MDT to create an accurate individualized treatment plan (17–20). This study has certain limitations. No clinical control study was performed and no comparison with previous cases was made to confirm the effect of MDT on the long-term prognosis of patients with cervical cancer. No life treatment survey was conducted to compare the effect of MDT on the quality of life. MDT for cervical cancer can significantly improve the immediate treatment outcome, but the effect on long-term prognosis, such as prolonging survival time and improving quality of life, needs further clinical observation. Therefore, more clinical trials should be conducted for active exploration (21, 22).

For the treatment of malignant tumors, a single discipline cannot fulfill the needs of the diagnosis and treatment of patients, and a simple subspecialty treatment system cannot provide comprehensive diagnosis and treatment advice to patients. Owing to the different levels of understanding and starting points of each specialist, they may give different or even opposite treatment opinions to patients on the basis of their professional point of view for the same disease. At the same time, clinical disciplines are gradually differentiating and refining, making it difficult for the discipline to keep abreast of the progress of other disciplines (17, 18). However, MDT is a face-to-face discussion and exchange of conditions of patients with malignant tumors through multidisciplinary meetings held at regular intervals. Thus, MDT can maximize the academic and professional advantages of multiple disciplines and make the best-individualized treatment plan that is most suitable for the patients on the basis of the treatment principles and clinical guidelines that are widely accepted (19, 20). MDT is gradually recognized by most countries after decades of development. Currently, several NCCN guidelines stipulate that all patients with diagnosed malignancies must undergo relevant MDT consultations before undergoing treatment (23, 24). The advantages of MDT are reflected in many aspects of the clinical consultation process such as interdepartmental cooperation, equal participation of patients and families, high-quality patient condition discussion, integration of relevant multidisciplinary, and education of young physicians. The MDT can provide better treatment for patients and improve their prognosis (25–27).

Accurate diagnoses and staging of tumors are important components of MDT for cervical cancer, and preoperative staging is determining whether the tumor should be treated with surgery, radiotherapy, or concurrent radiotherapy, and if surgery is selected, then the timing, surgical approach, and scope of surgery should also be determined (1–4). Postoperative staging can guide the way of postoperative adjuvant therapy such as radiotherapy, chemotherapy, or concurrent radiotherapy (28). Patients with advanced tumors can have PD-L1 mutation, the detection of which can guide different treatments of cervical cancer such as targeted treatment or immunization to determine the survival prognosis of patients and other conditions (29–35). If pregnant patients with cervical cancer choose to terminate their pregnancy, then the appropriate time to terminate the pregnancy and a reasonable and individualized treatment plan should be selected. If they choose to continue the pregnancy, then the safety of the fetus should be ensured, the prognosis of the mother should not be affected, and the effect of the treatment on the mother and fetus should be considered. For young patients, especially patients with cervical cancer, the preservation of reproductive functions is difficult during clinical diagnosis and treatment, and MDT can reflect the principle of individualized treatment while standardizing treatment to preserve the fertility of the patients while ensuring the therapeutic effect (36–39).

MDT is an inevitable trend in the development of tumor treatment. Moreover, it is also the development direction of cervical cancer treatment. MDT is patient-centered; therefore, instead of weighing the different opinions of physicians in each relevant department, multidisciplinary experts can work together to evaluate, discuss, and inform patients of the best diagnosis and treatment plan to achieve an integrated treatment for cervical cancer, which can simplify the medical consultation process, decrease clinical treatment time, improve treatment efficiency, save time and economic costs, and obtain a satisfactory medical experience (25–27, 40, 41). By discussing and arguing through interactive cooperation for treating patients, physicians can share the treatment strategy and clinical experience of top experts in various fields, update their professional knowledge, expand their treatment ideas, broaden their professional horizons, exchange the latest information, decrease the risk of errors, promote teamwork and disciplinary integration, cultivate new treatment ideas and techniques, create scientific research sparks, perform more relevant clinical research and basic research, and improve job satisfaction. Moreover, it can reduce interdepartmental referral rates, optimize and share resources, expand the influence of specialties, and improve the overall strength of hospitals (42, 43).

## Conclusion

The execution of MDT decisions is correlated with age; the younger the patients, the higher the execution efficiency. The patients who achieved MDT expectations through all implementation and partial implementation were correlated with age. The patients who achieved MDT expectations through all non-implementation did not correlate with age. The MDT plays a crucial role in clinical practice aspects, such as clinical staging, treatment plans, and whole treatment management of cervical cancer patients, which makes the treatment of cervical cancer more standardized, precise, and individualized. The MDT can significantly improve the recent treatment effect by improving long-term prognoses such as the survival time and quality of life of patients with cervical cancer. However, MDT requires further clinical observation and active exploration.

## Data availability statement

The raw data supporting the conclusions of this article will be made available by the authors, without undue reservation.

## Ethics statement

The present study was approved by the Ethical Committee of Ganzhou Cancer Hospital (2023002). The studies were conducted in accordance with the local legislation and institutional requirements. The participants provided their written informed consent to participate in this study.

Written informed consent was obtained from the individual(s) for the publication of any potentially identifiable images or data included in this article.

## Author contributions

JH completed most of the experiments, and XZ, HC, RL, and XW assisted in completing the experiments. YY, AC, DL, and CL completed data collection and statistics. JH and YP designed and supervised the completion of this experiment, and JH wrote this article. All authors contributed to the article and approved the submitted version.

## References

[1] Cohen PA Jhingran A Oaknin A Denny L. Cervical cancer. Lancet (2019) 393(10167):169–82. doi: 10.1016/S0140-6736(18)32470-X 30638582

[2] Buskwofie A David-West G Clare CA. A review of cervical cancer: incidence and disparities. J Natl Med Assoc (2020) 112(2):229–32. doi: 10.1016/j.jnma.2020.03.002 32278478

[3] Schaafsma M Plante M Mom CH van Trommel NE. Is less more in the surgical treatment of early-stage cervical cancer? Curr Opin Oncol (2022) 34(5):473–89. doi: 10.1097/CCO.0000000000000863 35880461

[4] Chargari C Peignaux K Escande A Renard S Lafond C Petit A. Haie-Méder C.Radiotherapy of cervical cancer. Cancer Radiother (2022) 26(1-2):298–308. doi: 10.1016/j.canrad.2021.11.009 34955418

[5] Sinyard RD Rentas CM Gunn EG Etheridge JC Robertson JM Gleason A. Smink DS.Managing a team in the operating room: The science of teamwork and non-technical skills for surgeons. Curr Probl Surg (2022) 59(7):101172. doi: 10.1016/j.cpsurg.2022.101172 35934411

[6] Bogani G Di Donato V Muzii L Casarin J Ghezzi F Malzoni M. Assessing the role of minimally invasive radical hysterectomy for early-stage cervical cancer. Eur J Obstet Gynecol Reprod Biol (2022) 275:64–9. doi: 10.1016/j.ejogrb.2022.06.004 35753229

[7] Chen Y Gong Y. Teamwork and patient safety in intensive care units: challenges and opportunities. Stud Health Technol Inform (2022) 290:469–73. doi: 10.3233/SHTI220120 PMC9201747 35673059

[8] Feng CH Mell LK Sharabi AB McHale M Mayadev JS. Immunotherapy with radiotherapy and chemoradiotherapy for cervical cancer. Semin Radiat Oncol (2020) 30(4):273–80. doi: 10.1016/j.semradonc.2020.05.003 32828383

[9] Avancini A Belluomini L Borsati A Riva ST Trestini I Tregnago D. Integrating supportive care into the multidisciplinary management of lung cancer: we can't wait any longer. Expert Rev Anticancer Ther (2022) 22(7):725–35. doi: 10.1080/14737140.2022.2082410 35608060

[10] Brown GTF Bekker HL Young AL. Quality and efficacy of Multidisciplinary Team (MDT) quality assessment tools and discussion checklists: a systematic review. BMC Cancer (2022) 22(1):286. doi: 10.1186/s12885-022-09369-8 35300636 PMC8928609

[11] Doose M Verhoeven D Sanchez JI Livinski AA Mollica M Chollette V. Team-based care for cancer survivors with comorbidities: A systematic review. J Healthc Qual (2022) 44(5):255–68. doi: 10.1097/JHQ.0000000000000354 PMC9429049 36036776

[12] Tran TH de Boer J Gyorki DE Krishnasamy M. Optimising the quality of multidisciplinary team meetings: A narrative review. Cancer Med (2022) 11(9):1965–71. doi: 10.1002/cam4.4432 PMC9089217 35257515

[13] Nwankwo C Shah R Shah A Corman S Kebede N. Treatment patterns and economic burden among newly diagnosed cervical and endometrial cancer patients. Future Oncol (2022) 18(8):965–77. doi: 10.2217/fon-2021-0727 35105169

[14] Friebel-Klingner TM Iyer HS Ramogola-Masire D Bazzett-Matabele L Monare B Seiphetlheng A. Evaluating the geographic distribution of cervical cancer patients presenting to a multidisciplinary gynecologic oncology clinic in Gaborone, Botswana. PloS One (2022) 17(8):e0271679. doi: 10.1371/journal.pone.0271679 35925976 PMC9352107

[15] Hitz F Ribi K Grote G Kolbe M Schmitz C Lamb BW. Team functioning across different tumour types: Insights from a Swiss cancer center using qualitative and quantitative methods. Cancer Rep (Hoboken) (2022) 5(8):e1541. doi: 10.1002/cnr2.1541 34582132 PMC9351662

[16] Soukup T Lamb BW Morbi A Shah NJ Bali A Asher V. Green JSA.Cancer multidisciplinary team meetings: impact of logistical challenges on communication and decision-making. BJS Open (2022) 6(4):zrac093. doi: 10.1093/bjsopen/zrac093 36029030 PMC9418925

[17] Saito Y Takahashi H Wakao F. Role of training the multidisciplinary oncology treatment team according to the Basic Plan to promote cancer control programs. Nihon Koshu Eisei Zasshi (2022) 69(7):527–35. doi: 10.11236/jph.21-128 35400729

[18] Eaton V Zambrano A Sanabria V Lopez R Kyei I Mra R. Innovative methodology for strengthening a multidisciplinary team approach in cities in low- and middle-income countries. JCO Glob Oncol (2022) 8:e2200149. doi: 10.1200/GO.22.00149 36252159 PMC9812446

[19] Guirado M Sanchez-Hernandez A Pijuan L Teixido C Gómez-Caamaño A Cilleruelo-Ramos Á. Quality indicators and excellence requirements for a multidisciplinary lung cancer tumor board by the Spanish Lung Cancer Group. Clin Transl Oncol (2022) 24(3):446–59. doi: 10.1007/s12094-021-02712-8 PMC8525055 34665437

[20] Zandee WT Merola E Poczkaj K de Mestier L Klümpen HJ Geboes K. Evaluation of multidisciplinary team decisions in neuroendocrine neoplasms: Impact of expert centres. Eur J Cancer Care (Engl) (2022) 31(6):e13639. doi: 10.1111/ecc.13639 35735226

[21] Park J Kim Y Kim J Kang S Kim K Kim JH. Health-related quality of life of patients with cervical cancer according to the duration of treatment and cancer progression. Asian Pac J Cancer Prev (2022) 23(6):1945–50. doi: 10.31557/APJCP.2022.23.6.1945 PMC9587850 35763635

[22] Friebel-Klingner TM Bazzett-Matabele L Ramogola-Masire D Monare B Ralefala TB Seiphetlheng A. Distance to multidisciplinary team clinic in gaborone, Botswana, and stage at cervical cancer presentation for women living with and without HIV. JCO Glob Oncol (2022) 8:e2200183. doi: 10.1200/GO.22.00183 36395437 PMC10166426

[23] Ajani JA D'Amico TA Bentrem DJ Chao J Cooke D Corvera C. Gastric cancer, version 2.2022, NCCN clinical practice guidelines in oncology. J Natl Compr Canc Netw (2022) 20(2):167–92. doi: 10.6004/jnccn.2022.0008 35130500

[24] von Mehren M Kane JM Agulnik M Bui MM Carr-Ascher J Choy E. Soft tissue sarcoma, version 2.2022, NCCN clinical practice guidelines in oncology. J Natl Compr Canc Netw (2022) 20(7):815–33. doi: 10.6004/jnccn.2022.0035 PMC10186762 35830886

[25] Layfield DM Flashman KG Benitez Majano S Senapati A Ball C Conti JA. Changing patterns of multidisciplinary team treatment, early mortality, and survival in colorectal cancer. BJS Open (2022) 6(5):zrac098. doi: 10.1093/bjsopen/zrac098 36254731 PMC9577547

[26] Harji DP Houston F Cutforth I Hawthornthwaite E McKigney N Sharpe A. The impact of multidisciplinary team decision-making in locally advanced and recurrent rectal cancer. Ann R Coll Surg Engl (2022) 104(8):611–7. doi: 10.1308/rcsann.2022.0045 PMC9680687 35639482

[27] Li JA Xu YL Ding N Ji Y Liu LX Rao SX. Pancreas multidisciplinary team optimizes the diagnosis and treatment of pancreas-related diseases and improves the prognosis of pancreatic cancer patients. Zhonghua Wai Ke Za Zhi (2022) 60(7):666–73. doi: 10.3760/cma.j.cn112139-20220408-00149 35775259

[28] Perrucci E Cerrotta A Macchia G Augurio A Campitelli M De Sanctis V. Postoperative treatment of intermediate-risk early stage cervical cancer: results of a survey from the Gynecology Study Group in the AIRO Gyn and MITO Groups. Crit Rev Oncol Hematol (2022) 174:103704. doi: 10.1016/j.critrevonc.2022.103704 35533816

[29] Mutlu L Tymon-Rosario J Harold J Menderes G. Targeted treatment options for the management of metastatic/persistent and recurrent cervical cancer. Expert Rev Anticancer Ther (2022) 22(6):633–45. doi: 10.1080/14737140.2022.2075348 35533682

[30] Čerina D Boraska Jelavić T Buljubašić Franić M Tomić K Bajić Ž Vrdoljak E. Is there a place for adjuvant chemotherapy in the treatment of locally advanced cervical cancer? Curr Oncol (2022) 29(8):5223–37. doi: 10.3390/curroncol29080415 PMC9332289 35892984

[31] Manrriquez EN Zakhour M Salani R. Precision medicine for cervical cancer. Curr Opin Obstet Gynecol (2022) 34(1):1–5. doi: 10.1097/GCO.0000000000000755 34596094

[32] Monk BJ Enomoto T Kast WM McCormack M Tan DSP Wu X. Integration of immunotherapy into treatment of cervical cancer: Recent data and ongoing trials. Cancer Treat Rev (2022) 106:102385. doi: 10.1016/j.ctrv.2022.102385 35413489 PMC10697630

[33] Liu MC Tewari KS. Current and emerging immunotherapies for recurrent cervical cancer. Clin Adv Hematol Oncol (2022) 20(2):108–15. 35120091

[34] Nishio S Yonemori K Usami T Minobe S Yunokawa M Iwata T. Pembrolizumab plus chemotherapy in Japanese patients with persistent, recurrent or metastatic cervical cancer: Results from KEYNOTE-826. Cancer Sci (2022) 113(11):3877–87. doi: 10.1111/cas.15479 PMC9633308 35792064

[35] Asakij T Khunnarong J Tangjitgamol S Rongsriyam K Tharavichitkul E Tovanabutra C. Salvage Treatment and Outcomes of Locally Advanced Cervical Cancer after Failed Concurrent Chemoradiation with or without Adjuvant Chemotherapy: Post Hoc Data Analysis from the ACTLACC Trial. Asian Pac J Cancer Prev (2022) 23(7):2263–9. doi: 10.31557/APJCP.2022.23.7.2263 PMC9727368 35901330

[36] Hammer A Haubjerg L Gibrael HS Winther A Pedersen LH Fuglsang K. Screening, diagnostics, and treatment of cervical cancer in pregnancy. Ugeskr Laeger (2022) 184(35):V03220187. 36065856

[37] Heimovaara JH Boere IA de Haan J van Calsteren K Amant F van Zuylen L. Ten-year experience of a national multidisciplinary tumour board for cancer and pregnancy in the Netherlands. Eur J Cancer (2022) 171:13–21. doi: 10.1016/j.ejca.2022.04.040 35696885

[38] Ronsini C Solazzo MC Bizzarri N Ambrosio D La Verde M Torella M. Fertility-sparing treatment for early-stage cervical cancer ≥ 2 cm: A problem with a thousand nuances-A systematic review of oncological outcomes. Ann Surg Oncol (2022) 29(13):8346–58. doi: 10.1245/s10434-022-12436-w PMC9640451 36064991

[39] Lenka F Jiří S. Current knowledge on fertilitysparing treatment of cervical cancer patients. Ceska Gynekol (2022) 87(5):362–70. doi: 10.48095/cccg2022362 36316219

[40] Kebede N Shah R Shah A Corman S Nwankwo C. Treatment patterns and economic burden among cervical and endometrial cancer patients newly initiating systemic therapy. Future Oncol (2022) 18(8):953–64. doi: 10.2217/fon-2021-0772 35094566

[41] Martini A Fallara G Pellegrino AA Nocera L Larcher A Raggi D. Multidisciplinary team referral at diagnosis for patients with non-metastatic renal cell carcinoma. Urol Oncol (2022) 40(8):384.e9–384.e14. doi: 10.1016/j.urolonc.2022.05.004 35667983

[42] Walraven JEW van der Hel OL van der Hoeven JJM Lemmens VEPP Verhoeven RHA Desar IME. Factors influencing the quality and functioning of oncological multidisciplinary team meetings: results of a systematic review. BMC Health Serv Res (2022) 22(1):829. doi: 10.1186/s12913-022-08112-0 35761282 PMC9238082

[43] Pillay B Wootten AC Crowe H Corcoran N Tran B Bowden P. The impact of multidisciplinary team meetings on patient assessment, management and outcomes in oncology settings: A systematic review of the literature. Cancer Treat Rev (2016) 42:56–72. doi: 10.1016/j.ctrv.2015.11.007 26643552

